# Feasibility, Safety, and Technical Success of the Flying Intervention Team in Acute Ischemic Stroke: Comparison of Interventions in Different Primary Stroke Centers with those in a Comprehensive Stroke Center

**DOI:** 10.1007/s00062-022-01220-8

**Published:** 2022-11-02

**Authors:** Alexander Kettner, Felix Schlachetzki, Tobias Boeckh-Behrens, Claus Zimmer, Silke Wunderlich, Frank Kraus, Roman Ludwig Haberl, Gordian Jan Hubert, Sandra Boy, Julia Henninger, Benjamin Friedrich, Christian Maegerlein

**Affiliations:** 1 Department of Diagnostic and Interventional Neuroradiology, Klinikum rechts der Isar, School of Medicine, Technical University Munich, Munich, Germany; 2 TEMPiS Telemedical Stroke Center, Department of Neurology, Center for Vascular Neurology and Intensive Care, University of Regensburg, Medbo Bezirksklinikum, Regensburg, Germany; 3 Department of Neurology, Klinikum rechts der Isar, School of Medicine, Technical University of Munich, Munich, Germany; 4 TEMPiS Telemedical Stroke Center, Department of Neurology, München Klinik Harlaching, Munich, Germany; 5 Department of Neurology, Asklepios Stadtklinik Bad Tölz, Bad Tölz, Germany; 6 Medical Department, Klinikum Dritter Orden, Munich, Germany; 7 Temedica GmbH, Munich, Germany

**Keywords:** Mechanical thrombectomy, Flying interventionalist, Helicopter, Helistroke, FIT

## Abstract

**Background:**

Prompt endovascular care of patients with ischemic stroke due to large vessel occlusion (LVO) remains a major challenge in rural regions as primary stroke centers (PSC) usually cannot provide neuro-interventional services. Objective The core content of the Flying Intervention Team (FIT) project is to perform thrombectomy on-site at a local PSC after the neuro-interventionalist has been transported via helicopter to the target hospital. An important and so far unanswered question is whether mechanical thrombectomy can be performed as safely and successfully on-site as in a specialized comprehensive stroke center (CSC).

**Methods:**

Comparison of 100 FIT thrombectomies on site in 14 different PSCs with 128 control thrombectomies at 1 CSC (79 drip-and-ship, 49 mothership) performed by a single interventionalist with respect to technical-procedural success parameters, procedural times, and complications.

**Results:**

There were no significant differences between the two groups in terms of technical success (95.0% successful interventions in FIT group vs. 94.5% in control group, *p* = 0.60) and complications (3% major complications in FIT vs. 1.6% in control group, *p* = 0.47). Regarding time from onset to groin puncture, there was no difference between FIT and the entire control group (182 vs. 183 min, *p* = 0.28), but a trend in favor of FIT compared with the drip-and-ship control subgroup (182 vs. 210 min, *p* = 0.096).

**Conclusions:**

Airborne neuro-interventional thrombectomy service is a feasible approach for rural regions. If performed by experienced neuro-interventionalists, technical success and complication rates are comparable to treatment in a specialized neuro-interventional department.

**Supplementary Information:**

The online version of this article (10.1007/s00062-022-01220-8) contains supplementary material, which is available to authorized users.

## Introduction

Mechanical thrombectomy (MT) has emerged as the guideline-based third pillar of optimal stroke treatment alongside professional neurological treatment on stroke units (SU) and intravenous thrombolysis (IVT) with recombinant tissue plasminogen activator [1–7]. In stroke patients suffering from intracranial large vessel occlusion (LVO), normally associated with high mortality and morbidity, MT with and without IVT has shown to be highly effective and superior to best medical treatment including IVT alone [8].

The introduction of IVT more than 20 years ago triggered the establishment of a dense network of SUs worldwide. Especially in rural regions, SUs were often realized by means of telemedical support from a comprehensive stroke center (CSC). The benefit of these telemedicine-assisted SUs was equivalent to regular SUs, at least in the prethrombectomy era [9, 10]. Thanks to a nationwide coverage also of rural regions with primary stroke centers (PSC), patient care could be substantially improved with the main goal of being able to initiate IVT as quickly as possible [7, 11].

After MT was established as a standard procedure, the treatment spectrum of on-site PSCs was no longer sufficient to provide complete care for stroke patients suffering from LVO.

**Fig. 1:**
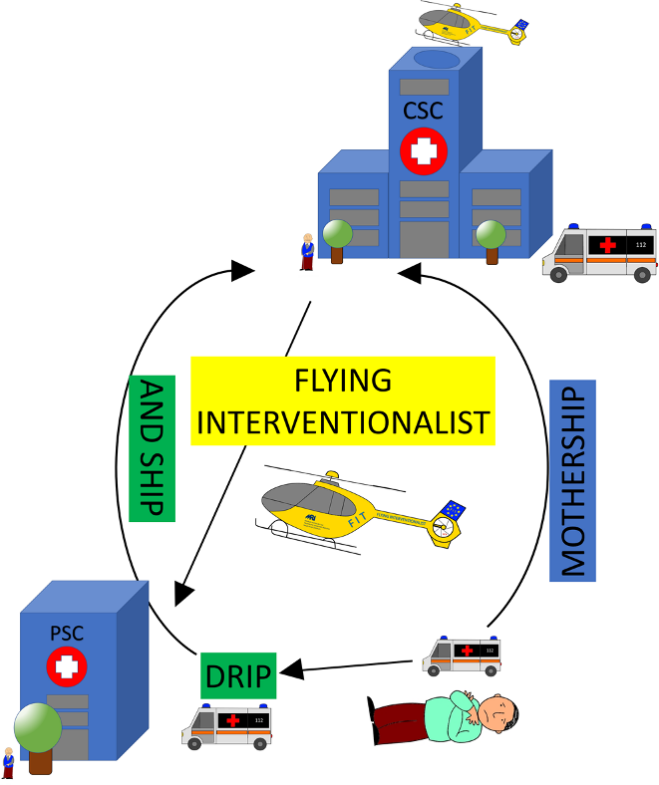
Schematic illustration of the three main care concepts in endovascular stroke therapy in rural areas. In the drip and ship model, the patient is first taken to the regional stroke unit where, if indicated, IVT is performed. In the case of an LVO, the patient is secondarily transferred to a CSC for thrombectomy. In the mothership model, the ambulance by-passes the regional stroke unit and drives directly to a CSC where all diagnostic and therapeutic options are available. In the Flying Intervention Team concepts, the patient receives IVT if indicated and is immediately prepared for MT on site in the event of an LVO. At the same time, the interventionalist is transported to the PSC to perform the endovascular procedure there

Therefore, patients suffering from LVO were increasingly transferred from a PSC to a CSC for MT secondary to locally initiated IVT [12]. Due to the sequence of treatments, this concept was later termed the drip and ship model (Fig. 1). The main advantage of the drip and ship model is that IVT can be started as soon as possible, and unnecessary transfer of patients not suffering from LVO, can be avoided; however, patients with ischemic stroke caused by LVO might benefit even more if allocated directly to CSCs for MT (termed the mothership model, Fig. 1). The main disadvantages of the mothership model are the possible later onset of IVT and the delay in the detection and treatment of intracranial hemorrhage (ICH) as another important stroke entity.

The application of clinical prehospital stroke scales has been proposed to triage in the mothership model; however, they are designed to identify any stroke but cannot sufficiently differentiate hemorrhagic from ischemic stroke, including LVO amenable to MT. A recent pilot study in the Baltimore metropolitan area demonstrated significantly faster initiation of MT (119 min) when being directly rerouted for MT to CSCs on application of the Los Angeles motor scale. The significantly faster initiation of MT showed a strong non-significant trend for better outcome, but also led to wrong allocation of patients in more than 50% [13].

Thus, the availability of MT is a fundamental infrastructural challenge potentially leading to an urban-rural divide in terms of quality of care for stroke patients. This is partially reflected in the fact that in rural areas in the USA MT is still conducted less frequently than in urban areas [14]. This might contribute to an overall 18% higher mortality of rural stroke patients [14].

Recently, driving or flying the interventionalist concepts were introduced as new approaches [15–17]. Here, MT is performed on site by bringing the neuro-interventionalist to the patient either by ground or air (Fig. 1). By parallelizing the preparation for MT and the transfer of the neuroradiologist shorter times from symptom onset to groin puncture and ultimately to reperfusion have been shown [15, 18]. Specifically, once an LVO is diagnosed on computed tomography (CT), CT angiography or magnetic resonance (MR) imaging, the patient can be transported directly to the local angiography suite or cardiac catheterization room, while the interventionalist is being transported to the destination hospital and the conditions for MT in the PSC, e.g., intubation, preparation of thrombectomy material, can be established additionally to immediate administration of IVT, if indicated.

However, the neuro-interventionalist may be confronted with new challenges under these circumstances. One crucial aspect is that angiographic systems in PSCs are probably different to the system the interventionalist is familiar with (Supplemental Table 3). Also, the PSC staff initially is not familiar with the Flying Intervention Team (FIT) and the kind of procedure which may affect the preparation of the set-up and specific requirements regarding anesthesia. In cases of intraprocedural complications. such as dissections or complicated vascular access, assistance by other experienced interventionalists is not available on site.

The primary purpose of this study therefore was to investigate whether EVT can be performed with equal procedural quality and without increased complication rates in the FIT setting compared to a high-volume CSC. In addition, the helicopter itself as a transport measure is evaluated for reliability and performance. To exclude factors derived from different levels of expertise of the neuro-interventionalist, all MTs in this study were performed by a single physician.

## Material and Methods

The approach of the FIT project has already been described previously [15, 18]. Briefly, the most important and innovative aspect of the project is that a helicopter is used exclusively to transport the neuro-interventionalist to the patient in the PSC. Neither the helicopter nor the helicopter team were engaged in other activities during the period of FIT service.

**Fig. 2:**
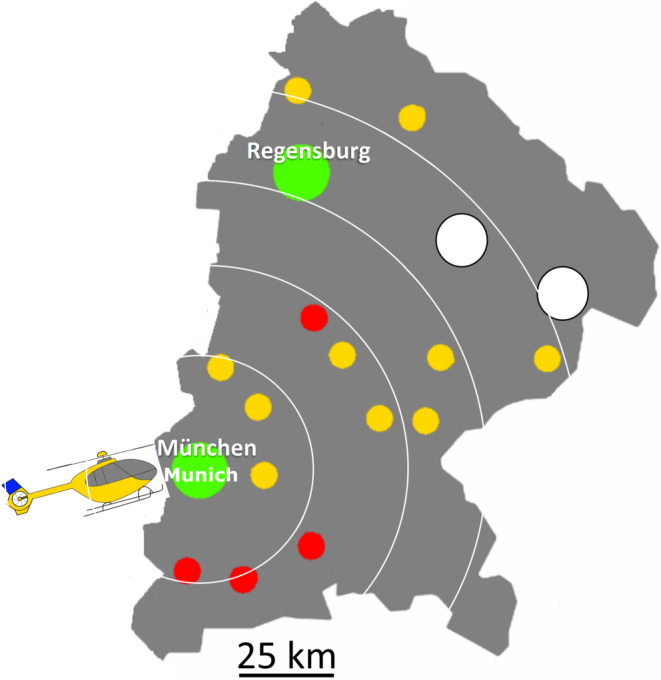
Illustration of the coverage area of the FIT service. Teleneurological headquarters were positioned in Munich and Regensburg. Interventional headquarters and place of departure for the FIT helicopter was Munich exclusively. Red dots represent PSCs with neurological departments, yellow dots represent PSCs with only internal medical departments. White dots represent CSC with neuro-interventional institutions that received patients for MT from PSCs (not within the scope of this study)

All weeks of the year were divided either into flight weeks (helicopter available) and transfer weeks (helicopter not available). Therefore, the FIT helicopter was available in 26 weeks of the year. The helicopter service had to be limited to the hours of 08:00 to 22:00. If LVO was diagnosed at a local PSC later than 22:00, patients had to be transferred to a CSC for MT (conversion to drip and ship model Fig. 1 and 2). Moreover, even during the flight weeks, there was always the option to transfer to the CSC (e.g., helicopter flight not possible due to poor sight).

This observational study focuses on the specific neuroradiological, interventional, and other technical aspects of the project including factors related to the helicopter itself. The FIT project was developed by the telemedical stroke network TEMPiS (Telemedizinisches Schlaganfallnetzwerk in Südostbayern). Coordination and operation and further development of the project was shared within a joint cooperation of the Department of Diagnostic and Interventional Neuroradiology, University Hospital rechts der Isar, Technical University Munich (TUM) and the telestroke network TEMPiS. TEMPiS was responsible for the neurological coordination of the project, while the University Hospital Munich TUM was responsible for the neuroradiological and interventional coordination of the project. Communication and activation of the FIT neuro-interventionalist was made by the TEMPiS neurologists while in transfer weeks patient transport was initiated by the local team of the PSC after decision for MT was made by the Interventional Neuroradiologist of University hospital together with the stroke neurologists. All procedures analyzed in this study were performed by a single interventional neuroradiologist who was also responsible for the interventional and neuroradiological coordination of the FIT project until November 2020. During the observation period of the study (February 2018 to November 2020), 69 further FIT procedures were performed by a total of four physicians different from the main interventionalist (3 physicians from München Klinik, MÜK, and 1 further physician from University hospital Munich TUM). To ensure optimal comparability of technical results between the two groups, these interventions were not included in the analysis.

All angiographic data were analyzed by the interventional neuroradiologist who performed the procedures and additionally by one further experienced interventional neuroradiologist with 7 years of experience in interventional stroke treatment. Evaluation of the modified thrombolysis in cerebral infarction (mTICI) score [19] as well as assessment of postinterventional embolization into new, previously unaffected vascular territory (ENT) as well as other interventional complications were determined by consensus between the two scientists.

### Initiation of the Project and Preparation of Material

Extensive preparations were required prior to project initiation. In total, the neuroradiological/interventional preparations took approximately 1 year until the first patient could be included on 15 February 2018.

In a first step, exploratory visits were made to the possible cooperation clinics within the Telemedicine Network TEMPiS to identify candidate PSC with appropriate local personnel and equipment for the project. Basic requirements by a collaborating hospital with PSC for participation in the project are listed in Supplemental Table 1.

**Fig. 3:**
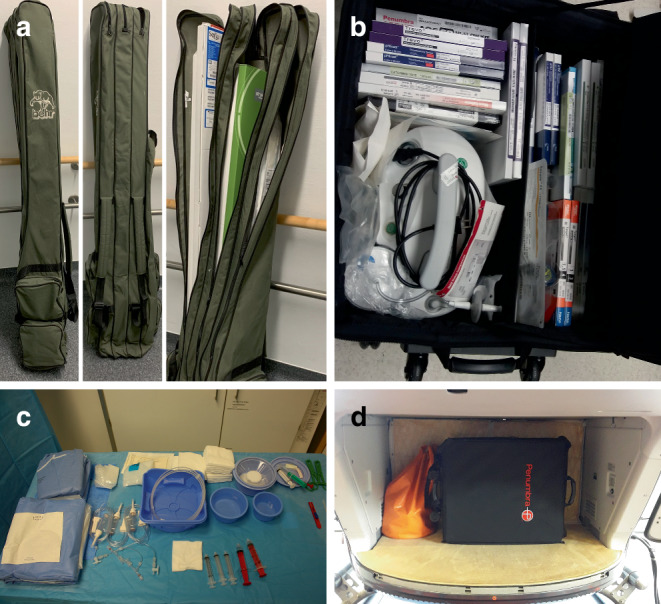
Illustration of the materials and the transport containers used in the project. Figure **a** shows the rod case with the long catheters. Figure **b** shows the wheeled suitcase opened with the materials and the aspiration pump inside. Figure **c** displays the materials that are stocked on site and have already been prepared by the local staff. Figure **d** presents the same case as shown in **b** loaded in the helicopter’s cargo hold

Special angio-sets were prepackaged and provided for the PSCs for preparation of the angio table prior to the arrival of the neuro-interventionalist. Next to some basic equipment like angio-sets, all relevant “neuro” materials needed for MT and complication management were brought by the interventionalist. A large-volume wheeled suitcase and a rod case (for the long catheters) were used for this purpose. Some of the material used in the project is depicted in Fig. 3. Replenishment of the local stock of angio-sets was provided by the FIT neuroradiologist. A notebook with Universal Mobile Telecommunications System (UMTS) reception was available to access CT or MR examinations from stroke patients while on air in order to be available for follow-up interventions without having to fly back to Munich. In case of multiple interventions, the angio-sets had to be sent by mail afterwards as only a single set was kept in the helicopter.

### Team and Training

On-site team training prior to project initiation was essential for the project. On the one hand, the on-site staff were trained on the basics of stroke as well as MT. Furthermore, the specific tasks of the local staff before, during and after MT were discussed in detail in order to make the cooperation with the FIT team as effective as possible. A critical content of the training sessions was the independent preparation of the angio-sets and anesthetic management of the patient prior to arrival of the neuro-interventionalist. Essential aspects of time and blood pressure management as well as questions regarding logistics and material storage were also covered. Only if the panel was unanimously convinced that treatment could be provided safely and professionally on site, was the respective PSC approved for FIT interventions on-site.

### Flying Intervention Team

The flying intervention team was composed of at least 4 persons. The missions were always accompanied by two Helicopter Emergency Medical Services (HEMS) helicopter pilots (either affiliated to helicopter company Helicopter Travel Munich GmbH (HTM) during the winter half-year or to helicopter company Allgemeiner Deutscher Automobil Club (ADAC) during the summer half-year), while the medical staff also consisted of two people. The interventionalist was assisted in all procedures by a long-term trained nurse or nurse practitioner (minimum experience of 3 years in neuro-interventions). In all interventions (flying and home), this assistant was actively involved in the interventions in a sterile manner and not as a circulating nurse.

During the corresponding weeks, the helicopter was available exclusively for the FIT project and was stationed either in Taufkirchen (near Munich) or in Munich. All flight missions started in Munich (Fig. 2).

During the interventions, the helicopter remained at the PSC helipad with the pilot team nearby to either bring the intervention team back to the CSC after the intervention was completed or to fly together to the next PSC for another intervention. Additionally, a circulating nurse was available for all procedures. This additional nurse was recruited from the on-site staffing pool both in the case of FIT missions and home interventions and was essentially responsible for delivering catheters and other thrombectomy materials.

### Inclusion Criteria

All patients who received MT by the neuroradiological coordinator himself were included in the study. In both groups, only patients with baseline angiographic findings requiring MT were included (see also Table 1). Patients that had an indication for MT initially but did not fulfill these indications in first DSA run (i.e. spontaneous recanalization, non-accessible occlusion location, etc.) were not included in this study. The indications for MT did not differ between the two groups and were in accordance with the current guideline recommendations of the societies of neurology and neuroradiology (see also Supplemental Table 2).

**Table 1:** Patient characteristics. Unless otherwise stated, the numerical values represent the median. The interquartile range is indicated in the parentheses

	Flying Interventionalist (*n* = 100)	Control (all, *n* = 128)		Drip and ship (*n* = 79)	Mothership (*n* = 49)	*p*-value
*Patient characteristics*						
Age (years, mean)	73.25 ± 12.86	75.40 ± 13.37		75.99 ± 12.42	74.61 ± 16.23	0.20 (all) 0.43 (DS) 0.46 (MS)
Female	62.0%	52.8%		52.6%	53.1%	0.23 (all) 0.21 (DS) 0.30 (MS)
*Baseline stroke data*						
NIHSS at admission	14 (8–17)	14 (9–18)		14 (10–19)	14 (8.75–18)	0.40 (all) 0.14 (DS) 0.80 (MS)
Wake-up stroke	28.0%	38.6%		35.4%	43.8%	0.145 (all) 0.38 (DS) 0.083 (MS)
IVT	61.0%	48.4%		53.2%	40.8%	**0.045 (all)** 0.39 (DS) **0.031 (MS)**
Occlusion site						
Prox. MCA (M1, prox. M2)	62.5%	61.7%		59.5%	61.2%	0.971
Carotid‑T	19.5%	19.5%		20.2%	18.4%	
Posterior circulation	12.5%	10.2%		11.4%	16.3%	
Other (e.g. ACA, dist. M2, M3)	5.5%	8.6%		8.9%	4.1%	
Tandem occlusion	10.0%	8.6%		7.6%	10.2%	0.871

*DS* Drip and Ship, *MS* mothership

### Patients

A total of 228 patients underwent MT, all performed by a single interventionalist between February 2018 and November 2020 either in one of the 14 FIT hospitals (intervention group, *n* = 100) or at University hospital Munich TUM (“home”, control group, *n* = 128).

An additional 69 FIT procedures were excluded as they were performed by other interventionalists during the same period. Also, additional 414 MT procedures were made at the University hospital Munich TUM by other interventionalists with the same time frame but were not included in the analysis.

Overall, all 228 procedures analyzed in this study were performed by a single neuroradiologist, ensuring good comparability of technical success and complication rates between the two groups.

### Workflow in the FIT Group

Pre-alert: if clinical appearance suggested a possible LVO, the interventionalist as well as the helicopter crew had been pre-alerted in this project. The pre-alert went into effect when the G‑FAST (Gaze, Face, Arm, Speech, Time) score reached 3 points. This was the case when three of the following four clinical criteria were pathological: gaze deviation, face weakness, arm weakness, speech problems [20]. In the event of a pre-alarm, a cascade was triggered at different levels. Thus, the interventionalist had to go immediately to the helipad of the University hospital Munich TUM. In the meantime, the helicopter team was checking the current weather conditions and possible problems regarding the targeted hospital. On site, the availability of the angio suite and capacities of the anesthesia department were checked. The final decision for or against an intervention was made on an interdisciplinary basis between the TEMPiS teleneurologist on call and the interventionalist. In the case of an FIT intervention, the patient was immediately transferred to the angio suite or cardiac catheter laboratory for preparation of the groins and the angio-set were prepared for the intervention. Meanwhile, the interventionalist was transported to the PSC to perform the procedure. Groin puncture and the procedure itself were performed by the interventionalist accompanied by a nurse. General anesthesia was defined as the anesthesiologic standard approach but procedures with the patient under local anesthesia were also permitted in justified cases.

### Workflow in the Control Group

In the control group, the interventionalist was alerted when a patient was diagnosed with LVO and was being transferred under the drip and ship model. Transportation was requested by the local PSC physician at the local rescue coordination center. The decision was made there whether to use ground transportation or helicopter transfer of the patient. It must be noted that PSCs in the transfer group are only partly identical to those in the FIT group. On average, PSCs in the control group are closer to the CSC (see Table 2) and do not all have telemedicine support from the same network as the FIT patients. During regular working hours, pre-alerting also took place in the control group in the CSC in the case of mothership patients (admission of a severely affected stroke patient, G‑FAST > 2). In such a case the angiography capacity was checked before CT/MRI and imminent elective procedures were eventually withheld until CT/MRI was finished. During duty hours, the interventionalist was contacted by telephone usually only if LVO had been diagnosed. In the control group, anesthesia was informed in every case only as soon as an intervention was indicated. The final decision for or against an intervention was made on an interdisciplinary basis between the stroke neurologists and the neuroradiologist. The interventionalist was responsible for deciding whether the procedure was performed with the patient under general or local anesthesia.

### Interventional Technique

Vascular access was preferably gained through the femoral artery. Selected interventions were also performed via a transradial or transbrachial approach or by direct puncture of the carotid artery. The interventionalist was free to choose the interventional technique. Methods comprised stent-retriever based and primarily aspirating maneuvers. Standard approach for anterior circulation LVOs were combination techniques using balloon-guided catheters, distal aspiration catheters and stent retrievers in combination [21, 22]. For posterior circulation LVOs standard approach was direct thrombus aspiration and/or combination techniques using distal aspiration catheters and stent retrievers.

### Evaluation of the Helicopter’s Operational Capability

The operational capability of the helicopter was assessed retrospectively by a participating helicopter pilot for the study observation period using daily flight reports from half of the flying weeks.

### Statistical Analysis

Statistical analyses were performed using SPSS software version 28 (IBM, Armonk, NY, USA). Differences between the two groups were compared using the Mann-Whitney U‑test or Fisher’s exact test depending on the type of variables analyzed. Statistical significance was assumed at *P* < 0.05.

### Data

The data of this study were gathered from two different registries. Data for the FIT group were collected in the FIT registry. Data for the control group were collected from an individual registry of the University hospital Munich TUM.

## Results

The two groups (FIT vs. control) did not differ regarding the patients age, sex, stroke severity (NIHSS), and occlusion site. The rate of bridging thrombolysis was higher among FIT patients (61.0% vs. 48.3%, *p* = 0.045), Table 1. There was no difference regarding interventional treatment success like necessary maneuvers, postinterventional reperfusion (95.0% TICI 2b/3 reperfusion in FIT vs. 94.5% TICI 2b/3 in control group, *p* = 0.60), and periinterventional complications. Major complications were 3.0% in HELISTROKE and 1.6% in control group (*p* = 0.473). Regarding onset to groin times, there was neither a difference between FIT and the entire control group (182 vs. 183 min, *p* = 0.28), nor a significant difference between FIT and the drip and ship control subgroup (182 vs. 210 min, *p* = 0.096). Onset-to-groin times were shorter for mothership patients than for FIT (135 min vs. 182 min, *p* < 0.01). See also Table 2 for further details.

**Table 2:** Technical and procedural outcome parameters. Unless otherwise stated, the numerical values represent the median. The interquartile range is indicated in the parentheses

	Flying Interventionalist (*n* = 100)	Control (all, *n* = 128)		Drip and Ship (*n* = 79)	Mothership (*n* = 49)	*p*-value
*Distance*						
Distance to CSC in kilometres	50 (34.5–64.5)	–		36 (11; 50)	–	**< 0.01 (DS)**
*Times*						
Onset to groin puncture in minutes	182 (159; 240)	183 (140; 239)		210 (180; 255)	135 (1; 165)	0.28 (all) 0.096 (DS) **< 0.01 (MS)**
Groin puncture to recanalization in minutes	37 (25; 52)	25 (18; 47)		33 (19; 46)	21 (17; 49)	0.28 (all) 0.34 (DS) 0.11 (MS)
*Arterial access site*						
Femoral	92.0%	89.8%		92.4%	85.7%	0.87
Radial/Brachial	4.0%	7.0%		6.3%	8.2%	
Carotid Direct Puncture	4.0%	3.1%		1.3%	6.1%	
*Postinterventional mTICI*						
mTICI 3	69.0%	48.4%		49.4%	47.0%	0.60
mTICI 2c	3.0%	7.0%		7.6%	6.1%	
mTICI ≥ 2b	95.0%	94.5%		94.9%	93.9%	
Number of maneuvers	1 (1; 2)	1 (1; 2)		1 (1; 2)	1 (1; 2)	0.87
*Major Complications*						
All	3%	1.6%		1.3%	2.0%	0.47
Vessel perforation	1%	0%		1.3%	0%	
Dissection (flow restricting)	1%	0.8%		0%	2.0%	
ENT	1%	0.8%		0%	0%	
*Radiation exposure*						
Dose area product (Gy cm^2^)	50 (30; 85)	81 (40; 147)		82.24 (47.40; 140.50)	77.65 (33.64; 160.25)	**<** **0.01**

*DS* Drip and Ship, *MS* mothership

The dose area product was significantly lower in the FIT group than in the control group (50 Gy × cm^2^ vs. 81 Gy × cm^2^, *p* = 0.01), see also Table 2.

The evaluation of the helicopter’s operational capability showed that 89% of the intended missions could be carried out as planned with the mission helicopter during the study period. Due to poor sight conditions, one mission was only possible thanks to instrument flight. This technique, for which the helicopter team also requires special licenses, was not a standard procedure; however, the flights were otherwise conducted on sight. Patients that could not be flown were transferred by conventional ground-based means to the nearest CSC for MT. On five missions, the PSC was reached according to plan by the FIT team, but the helicopter then had to make the return flight before the procedure was completed. The medical team then had to be transported back to CSC by taxi in these cases.

## Discussion

The most important finding of the observational study presented here is that FIT interventions can be performed as successfully at the various PSCs as at a highly specialized university hospital. This includes not only the reperfusion rates but also the procedure times as well as the periprocedural complications. These aspects were not addressed in detail in the preceding FIT publication [18] but is a controversial issue in the neuro-interventional community.

The higher rate of IVT in the FIT group might be explained by different indications for IVT in the extended time window in both groups, and different selection criteria made by the telemedical stroke neurologist. While IVT was not administered in the CSC (control group) in the extended time window if MT was indicated according to the Wake-Up trial exclusion criteria [23], in several (high-volume) PSCs the indication for IVT was made on the basis of CT perfusion independent of the indications of successive MT. Also, considering the recently published RACECAT trial, in which a lower rate of IVT was performed in the mothership cohort [24], the FIT project may have the advantage of ensuring a high rate of IVT despite rapid initiation of MT.

It is questionable if the more frequent IVT treatments in the FIT group due to partially different approaches between both groups may have had an impact on the final reperfusion results. While previous studies provided inconsistent findings on whether IVT in addition to MT provided benefit [25–27], the SWIFT-DIRECT study is now the first to demonstrate the benefit of IVT in addition to MT for patients suffering an LVO without contraindications against IVT [28]. Moreover, the rate of successful (TICI 2b-3) reperfusions in this study was significantly higher in the IVT + MT group than in the MT group (96% vs. 91%, *p* = 0.047). Although there was only a trend regarding TICI3 outcomes, there was at least a 3% higher rate of TICI3 reperfusions in the combination group [28].

In addition, the difference in TICI3 results may be, at least in part, a technical artifact, because individual, very distal vessel occlusions may not have been detected by the two readers because the image quality of some angiography systems in the PSCs lags behind the image quality of the dedicated biplane neuroangiography system in the University hospital Munich TUM. Therefore, the mTICI 2b rates are regarded to be more comparable between both groups as remaining large branch occlusions are reliably visible also on the less sophisticated local angiography machines. As the rate of successful recanalization (TICI2b-3) was the same in both groups and there are technical uncertainties described above, the procedural outcome is thus assumed to be equivalent.

Given the immense effort that went into the FIT project, it may seem surprising that no significant difference was seen in terms of time from symptom onset to groin puncture; however, it was not the intention of the present study to shed light on the question of a time advantage of the FIT concept, since this question could already be answered in advance in terms of a significant time advantage of the FIT project over the drip and ship model before [18]. There are different reasons for the discrepancy in the present study.

First, the PSCs in the control group from which patients were transferred to the CSC were not identical to those in the FIT group. Therefore, distances to the CSC were different (Table 2). In addition, hospitals that are not part of the same network may have different structures and procedures than network hospitals.

Second, the procedures in the University hospital Munich TUM are designed for the fastest possible door to groin time. For example, drip and ship patients usually do not receive repeat imaging in the CSC but are taken directly to angio-suite if neurologically stable. Only if there is a significant change in clinical symptoms when the patient arrives at the CSC, possibly CT, CTA and CTP are being repeated. On the other hand, because the main outcome was technical feasibility and to gain a larger control group the comparison group is composed not only of drip and ship patients but also of patients primarily admitted to the CSC (mothership patients). In the subanalysis comparing the FIT patients with the drip and ship patients exclusively, there is a trend towards a shorter time from symptom onset to groin puncture in favor of the FIT patients. In the analysis of FIT vs mothership patients, a time advantage was found in favor of the mothership group; however, a difference of merely 47 min for coverage of the rural population compared to the urban population seems quite small given the distances that had to be covered under the FIT project.

The finding that less amount of radiation was applied in the FIT group is in-line with an earlier study comparing endovascular stroke treatment on single plane versus biplane angiography systems [29]. The reason for this is primarily that there are only single plane angiography systems in the PSCs and a biplane angiography system in the CSC. At the biplane angiography system, twice the amount of DSA images is generated per DSA run. Interventions at biplane angiography systems formally offer more safety, since the probability of the material being accidentally navigated into the wrong vessel and causing damage there is lower. An example would be accidental probing of the anterior choroidal artery while probing the distal internal carotid artery. This could in principle lead to injury of this tiny vessel; however, in the study performed here, there were no increased complications at the single-plane machines of the PSC’s.

The helicopter is basically an extremely fast means of transport. The Eurocopter 135 used in this project is a light twin-engine helicopter with a travel speed of approximately 230 km/h. The pure flight time is therefore significantly less than the time taken by a ground-based transport. On the other hand, a possible disadvantage is that it can usually only be flown on sight. In very poor visibility conditions, caused for example by fog, it is not possible to fly the helicopter [30]. Also, thunderstorms and hail are to be avoided [31]. This is important, not least because HEMS missions are associated with a significantly increased risk of catastrophic accidents compared to other emergency medical services (EMS) [32, 33]. Safety concerns of the helicopter pilot concerning the weather situation resulted in a failure rate of 11% in the FIT group in this study meaning that of a total of 112 patients that met the inclusion criteria only 100 could be treated on site and 12 patients had to be transferred to a CSC for MT instead of being treated on site. In addition, the failure rate of helicopter missions is known to be even significantly higher during the night hours [34]. Since the FIT project did not envisage any operations between 22:00 and 08:00, it can be assumed that the failure rate is likely to be higher than the 11% in the case of 24/7 coverage. Additionally, the flight preparation phase until lift-off is significantly longer during night hours which might reduce the time advantage compared to a ground-based transportation [18, 35].

Another critical issue concerning the helicopter is the fact that the intervention team must present itself at the CSC helipad for take-off. There may be a timely disadvantage if the team is not in the hospital at the time of the alert, especially if there is no pre-alert. Direct ground-based transport of the intervention team to the PSC without going through the CSC may be faster here, depending on the distance to the PSC in question. Ultimately, the maximum time advantage for the helicopter occurs when the FIT team is available 24/7 at the CSC as part of a presence service. The abovementioned points may speak in favor of an additional ground-based system if the helicopter is not ready for operation. This may increase the number of stroke patients who benefit from on-site MT. With this methodological modification the number of stroke patients who benefit from on-site thrombectomy might be increased.

In this context, decentralized multicenter neuroradiological participation in MT services could also be discussed, in which each of the CSCs provide a mobile service for their closest PSCs. The discussion of this is beyond the scope of this paper and should be the subject of further research.

Our study has several limitations. Angiographic results were carefully evaluated by the interventionalist in consensus with a second experienced interventional neuroradiologist from the department of neuroradiology; however, it seems likely that the rate of mTICI 3 reperfusions in the FIT group was overestimated. This might be an effect of the inferior image quality of the local angiography machines/mobile C‑arm in comparison to the standard of a high-end biplane neuroangiography system. Very small branch occlusions might have been missed in several cases. Nevertheless, in our view, there is a relatively high degree of certainty regarding the interpretation of large branch occlusions. Thus, the number of “successful” interventions (> mTICI 2b) should be reliable. In our view, even a core laboratory could not have adequately addressed this technical weakness.

Another uncertainty concerns the statement that the technical successes as well as the complication rates are similar in both groups. Overall, the interventions in the peripheral PSCs are disproportionately more demanding than in the CSC. This project requires a highly experienced neuro-interventionalist who can cover the full spectrum of modern interventional neuroradiology including innovative complication management with limited material on site. If the interventions are performed by less experienced physicians, different results may be seen. In the CSC, there are also other experienced colleagues available who can provide support. This is not possible in FIT interventions.

Also, the data of the helicopter availability were gathered from only half of the flying weeks during the study period only representing one of the two helicopter companies. Therefore, these results must be interpreted with care.

Considerable financial and personnel resources were made available for the project. Although the results are encouraging, the question arises as to whether and to what extent such a project could be transferred to standard care. This is ultimately defined not only by limited financial resources but also by the scarcity of experienced interventional neuroradiologists. Regarding the cost-effectiveness of the project, further scientific studies are currently being conducted.

PSCs in both groups were not identical, therefore, the comparison of time delays between FIT and the control group is of limited value because the focus of the study was on the technical and procedural comparison of the concepts. We have deliberately chosen to highlight the technical aspects, since clinical comparability is limited due to the different patient collectives.

In addition, post-interventional management in CSC is normally performed in intensive care units or in SU with high experience. Early versus late extubation, blood pressure management and detection of early reocclusion or hyperperfusion syndromes are additional factors contributing to neurological outcome. Especially in hospitals that do not have a neurological department (see Supplemental Table 3), it must be ensured that neurological and neuroradiological expertise would be available at all times after the interventions. The potential difference of postinterventional neurocritical care of complicated neurovascular patients in specialized CSCs versus telemedical PSCs are an area ill investigated to date. This important issue was attempted to be addressed in the present project by dedicated quality management including specific SOPs, standardized teleconsultations for follow-up, and 24/7 availability of the interventional neuroradiologist as well as the stroke neurologist on call.

## Conclusion

The Flying Intervention Team (FIT) thrombectomy service is a feasible approach for rural regions. If performed by experienced neuro-interventionalists, technical success and complication rates are similar to treatments in a specialized neuro-interventional department. This concept could be integrated into existing care concepts, including ground-based transport of interventionalists, and could reduce the number of secondary patient transports with the associated reduced time delays.

## Supplementary Information

Supplemental Table 1 General requirements for a primary stroke center to participate in the FIT project. *HEMS* Helicopter Emergency Medical Services

Supplemental Table 2: Clinical and imaging inclusion criteria within the study. *MeVO* Medium Vessel Occlusions, *ACA* Anterior Cerebral Artery, *BO* Basilar Artery Occlusion, *ASPECTS* Alberta Stroke Program Early CT Score, *mRS* modified Rankin Scale

Supplemental Table 3 overview of FIT hospitals, distances to CSC, local responsibilities, and angio systems. *PSC* Primary Stroke Center, *NL* neurology, *MED* internal medicine

## Abbreviations

CSC: Comprehensive stroke center
CT: Computed tomography
DS: Drip-and-ship
DSA: Digital subtraction angiography
ENT: Embolization to new territory
EVT: Endovascular treatment
FIT: Flying Intervention Team
ICH: Intracranial hemorrhage
IVT: Intravenous thrombolysis
LVO: Large vessel occlusion
MR: Magnetic resonance
MS: Mothership
MT: Mechanical thrombectomy
NIHSS: National Institutes of Health Stroke Scale
PSC: Primary stroke center
SU: Stroke unit
TEMPiS: Telemedizinisches Schlaganfallnetzwerk Südostbayern
TUM: Technical University Munich
UMTS: Universal Mobile Telecommunications System
