# Supplementary material for: Feasibility, Safety, and Technical Success of the Flying Intervention Team in Acute Ischemic Stroke: Comparison of Interventions in Different Primary Stroke Centers with those in a Comprehensive Stroke Center
Source: Clin Neuroradiol. 2022 Nov 2;33(2):393–404. doi: 10.1007/s00062-022-01220-8 (PMC10219878; doi:10.1007/s00062-022-01220-8)
Supplement: Supplementary file 1 — Supplemental Table 1 General requirements for a primary stroke center to participate in the FIT project. HEMS Helicopter Emergency Medical Services [file 62_2022_1220_MOESM1_ESM.docx]

Supplemental Table 1 General requirements for a Primary Stroke Center to participate in the HELISTROKE project. HEMS, Helicopter Emergency Medical Services

| General Infrastructure | Imaging | Intervention |
| --- | --- | --- |
| Telemedical participation in the network (either neurological or internal medicine department in responsibility of the local Stroke Unit)  Intensive Care Unit obligatory | CT and CTA availability 24/7; image quality of CT/ CTA was assessed for every participating PSC before final approval  CT Perfusion necessary for patients in delayed time window (>6h), optional | Any X-ray system with DSA-function on-site  Image quality of X-ray fluoroscopy and DSA was approved for every participating PSC before final approval |
| Helipad nearby hospital compliant with regulations of HEMS | MRI available on-site; MR examinations must be available at least on weekdays/ working hours. | Adequate on-site storage capacities near intervention room for materials such as Angio-Sets, Pressure bags etc. |
