# Supplementary material for: Feasibility, Safety, and Technical Success of the Flying Intervention Team in Acute Ischemic Stroke: Comparison of Interventions in Different Primary Stroke Centers with those in a Comprehensive Stroke Center
Source: Clin Neuroradiol. 2022 Nov 2;33(2):393–404. doi: 10.1007/s00062-022-01220-8 (PMC10219878; doi:10.1007/s00062-022-01220-8)
Supplement: Supplementary file 2 — Supplemental Table 2: Clinical and imaging inclusion criteria within the study. MeVO Medium Vessel Occlusions, ACA Anterior Cerebral Artery, BO Basilar Artery Occlusion, ASPECTS Alberta Stroke Program Early CT Score, mRS modified Rankin Scale [file 62_2022_1220_MOESM2_ESM.docx]

Supplemental Table 2: Clinical and imaging inclusion criteria within the study. MeVO, Medium Vessel Occlusions; ACA, Anterior Cerebral Artery; BO, Basilar Artery Occlusion; ASPECTS, Alberta Stroke Program Early CT Score; mRS, modified Rankin Scale

| Clinical Indications | Imaging Indications |
| --- | --- |
| Clinical diagnosis of acute (ischemic) stroke | Large Vessel occlusion responsible for clinical deficit (e.g. intracranial ICA, M1, M2, segment of MCA, BA), tandem occlusions;  singular MeVO (ACA, M3 etc.) |
| Neurological deficit relevant to daily life | Occlusion site probably reachable with thrombectomy device |
| Symptom onset <24h, if symptom onset>6h extended imaging (perfusion imaging) necessary (anterior circulation) | ASCPECTS > 5 (anterior circulation)  Exclusion of acute cerebral hemorrhage |
| In case of BO: onset <24, no extended imaging necessary |  |
| Premorbid mRS <4 |  |
