# Supplementary material for: Feasibility, Safety, and Technical Success of the Flying Intervention Team in Acute Ischemic Stroke: Comparison of Interventions in Different Primary Stroke Centers with those in a Comprehensive Stroke Center
Source: Clin Neuroradiol. 2022 Nov 2;33(2):393–404. doi: 10.1007/s00062-022-01220-8 (PMC10219878; doi:10.1007/s00062-022-01220-8)
Supplement: Supplementary file 3 — Supplemental Table 3 overview of FIT hospitals, distances to CSC, local responsibilities, and angio systems. PSC Primary Stroke Center, NL neurology, MED internal medicine [file 62_2022_1220_MOESM3_ESM.docx]

Supplemental Table 3 overview of HELISTROKE hospitals, distances to CSC, local responsibilities, and angio systems; PSC, Primary Stroke Center; NL, Neurology; MED, Internal Medicine

| FIT-PSC | Number of patients | Distance to CSC [km] | Medical discipline of local PSC | Angiography system/ machine |
| --- | --- | --- | --- | --- |
| AG | 11 | 44.2 | NL | ArtisZee MP monoplane, Siemens |
| AÖ (since 12/2019) | 2 | 80.5 | MED | ArtisZee monoplane, Siemens |
| TÖL | 10 | 42.4 | NL | AXIOM Artis monoplane MP, Siemens |
| BUL | 2 | 122.9 | MED | Mobile C-arm, Ziehm Vison R (until July 2020); Innova 2000, GE (since July 2020) |
| CHA | 5 | 144.0 | MED | ArtisOne AXN monoplane, Siemens |
| EBE | 5 | 27.5 | MED | Allura Xper FD 20 cardio monoplane, Philips |
| EG | 3 | 91.1 | MED | Allura Xper FD 20 monoplane, Philips |
| ED | 13 | 28.0 | MED | Artis Zeego monoplane, Siemens |
| FS | 7 | 31.7 | MED | Allura Xper FD 20, Philips |
| LA (since 12/2019) | 1 | 59.8 | NL | ArtisZee monoplane, Siemens |
| MÜ | 4 | 70.1 | MED | ArtisOne, AXN monoplane, Siemens |
| RO | 26 | 50.0 | NL | 1.ArtisZee ceiling monoplane, Siemens; 2.ArtisZee multi-purpose, Siemens |
| ROT | 5 | 121.0 | MED | Allura Integris Xper FD 10, Philips |
| VIL | 6 | 65.9 | MED | ArtisZee floor, Siemens |
| CSC Control | 128 | 0 | NL | Azurion 7 biplane 20/20, Philips |
